# The effect of visual feedback on balance rehabilitation in people with impaired ankle instability: a systematic review and meta-analysis

**DOI:** 10.1186/s13102-024-01041-x

**Published:** 2025-04-10

**Authors:** Chuan qiu shui Wang, Kun Wang, Liang Sun, Shi qi Liu, Jiong Luo

**Affiliations:** 1 School of Physical Education, Sports Rehabilitation Center, Southwestern University, Chongqing, China; 2 College of Physical Education, Southwest University, Beibei District, Chongqing, China

**Keywords:** Augmented Feedback, Balance Exercise, Virtual Reality, The Star Excursion Test, Visualization

## Abstract

**Background:**

Ankle instability is a consequence of ankle injuries that can impair balance and affect an individual’s ability to perform activities of daily living. Consequently, visual feedback is frequently employed in rehabilitation to enhance training outcomes. However, inconsistencies exist in the scientific literature regarding the effect size of visual feedback and the optimal method of delivery.

**Aim:**

The objective of this systematic review and meta-analysis was twofold: firstly, to conduct a comprehensive assessment of the potential impact of visual feedback on balance exercises for individuals with ankle instability; secondly, to quantify the effects of visual feedback on balance in this population.

**Methods:**

A total of 10 studies were included in this systematic review and meta-analysis. The review was conducted in accordance with the PERSiST (Implementing Prisma in Exercise, Rehabilitation, Sport Medicine, and Sports Science) guidance. The research was conducted using four databases and was required to be written in English. Additionally, the studies had to involve the provision of visual feedback during balanced rehabilitation.

**Results:**

The provision of visual feedback has been demonstrated to enhance the efficacy of rehabilitation programs designed to improve balance. Improvement in static, dynamic, and perceptual balance during balance training. The provision of visual feedback was found to simultaneously increase the subjects’ subjective motivation to train and their satisfaction and enjoyment of the rehabilitation training. The results demonstrated that visual feedback improved the Foot and Ankle Ability Measure by approximately 17% (MD = 2.42, 95%CI = 0.72 to 4.12, I²[total] = 0%). Additionally, the provision of visual feedback during the training cycle may positively affect the star excursion balance test (SEBT) (MD = 4.83, 95%CI = 3.09 to 6.56, I²[total] = 21%). Moreover, the Biodex system is expected to demonstrate notable improvements in measures of balance (MD = 0.14, 95% CI = 0.01 to 0.28, I²[total] = 24%).

**Conclusion:**

The provision of visual feedback in the context of balance rehabilitation has been demonstrated to facilitate beneficial adaptations to balance in individuals presenting with ankle instability. The research included in our analysis demonstrates the positive impact of visual feedback, with nearly all results exhibiting superior outcomes relative to those observed in the absence of visual feedback. Visual feedback can be employed as an “additive” or alternative to conventional rehabilitation for individuals with ankle instability.

**Supplementary Information:**

The online version contains supplementary material available at 10.1186/s13102-024-01041-x.

## Introduction

Ankle sprains represent a significant proportion of skeletal injuries associated with sports, accounting for approximately 15% of all sports-related injuries [1]. Ankle structures and proprioceptive systems are affected by recurrent ankle sprains, mechanical laxity, and perceived instability, which is typically described as a sensation of the ankle joint “giving way“ [2]. A paucity of attention devoted to rehabilitation following ankle sprains in a considerable number of patients has been identified as a significant contributing factor to the high incidence of injury recurrence and an elevated risk of developing ankle dysfunction, including functional ankle instability (FAI) and chronic ankle instability (CAI) [3, 4]. Functional ankle instability is usually defined as temporary or reversible ankle instability resulting from an ankle injury and typically develops in 40% of patients after an ankle sprain, manifesting as impairment of some motor functions of the ankle joint and reduced performance on some balance tests [5, 6]. Chronic ankle instability is usually defined as a long-term injury caused by repetitive ankle sprains, which is more common in sports and is associated with ‘perceptual instability’ and ‘mechanical instability’. Functional ankle instability can be one of the causes of long-term recurrent ankle sprains [7–9]. Ankle instability has been demonstrated to result in impaired ligament fiber integrity and broken joint movement patterns [10, 11]. Furthermore, balance and dorsiflexion range of motion were found to be diminished in individuals with impaired mobility [1, 12]. The aforementioned effects may elevate the probability of tripping, falling, or reinjuring the ankle, with long-term deleterious consequences on quality of life and the capacity to perform activities of daily living. Consequently, rehabilitative exercises are imperative for individuals with ankle instability [13].

Prior research has indicated that the efficacy and patient satisfaction of conventional clinical diagnostic and surgical treatments may be suboptimal [14]. In light of the available therapeutic options, there has been a focus on the prevention of ankle injuries and the rehabilitation of ankle instability [15, 16]. Researchers are engaged in ongoing investigations into the efficacy and cost-effectiveness of rehabilitation methodologies, encompassing stability-based balance exercises, strength training, neurofeedback training, and multimodal training. These methods have been demonstrated to be effective in improving balance and postural control in patients with ankle injuries and to have a positive effect on the recovery of ankle functional mobility [17–20].

Incorporating augmentative feedback (AF) into conventional training regimens is thought to enhance the efficacy of both training and rehabilitation, particularly in acute athletic performance and chronic adaptations [21]. To illustrate, in the context of augmentative feedback, the provision of visual feedback may prove more efficacious than verbal feedback in enhancing the velocity and power output of participants engaged in resistance exercises [22]. The provision of visual feedback enables the generation of real-time data through the utilization of visualization devices that accurately detect motion in controlled environments. This allows for the accurate representation of the user’s true movement, while simultaneously providing information for the trainer or physician to gather [23]. Several studies have previously employed visual feedback as a tool for facilitating balance exercises. For example, the use of virtual devices as an intervention tool has been demonstrated to be an effective method for improving balance function in patients who have suffered a stroke or who have musculoskeletal injuries or amputations affecting the lower extremities [24, 25]. Furthermore, visual feedback training (VFT), which integrates conventional balance exercise training with digital information display, has been demonstrated to enhance knee functional recovery and motor control in patients who have undergone knee arthroplasty [26, 27]. The utilization of diverse active visual screen-based motor games offers visual feedback through an array of visual perceptual processing challenges, thereby prompting lower limb muscle responses that enhance postural control and balance [28]. In light of the evidence indicating that visual feedback is an effective method for enhancing performance on the Posterior Lateral Star Excursion Balance Test (SEBT), it may serve as a valuable tool for improving dynamic postural control and balance exercises in clinical rehabilitation and athlete training [29].

Consequently, several studies have examined the impact of visual feedback on individuals with ankle functional instability [30–32]. The data suggest that beneficial improvements in dynamic balance, static balance, and foot and ankle functionality were more significant in patients when visual feedback was provided during balance exercises. For instance, Kim’s study demonstrated that a virtual reality program (VR) enhanced dynamic balance more effectively than static balance in patients with functional ankle instability (FAI) [30]. Although the intervention’s efficacy was validated, the studies faced limitations due to their relatively small sample sizes and the inconsistencies in the indicators used to assess balance ability. Punt demonstrated that the Wii Fit effectively improved foot and ankle ability scores while reducing pain sensation during walking in patients with ankle sprains. However, the findings suggested that it was not as effective as other forms of physical therapy or exercise therapy [31]. To our knowledge, there is currently a deficiency of meta-analyses that investigate the effects of visual feedback on balance rehabilitation in individuals with ankle instability. Given this gap, it is crucial to ascertain whether visual feedback can yield a significant positive impact on balance rehabilitation for those with ankle instability, particularly in comparison to control or placebo groups. Accordingly, we conducted a systematic review and meta-analysis in accordance with the PERSiST guidelines and the PRISMA statement. Our objective was to evaluate the effects of visual feedback on enhancing ankle function and improving balance outcomes in individuals with ankle instability.

## Methods

### Information sources and search strategy

This systematic review and meta-analysis had two primary objectives: (1) to perform a thorough evaluation of the effects of visual feedback on balance exercises in individuals with ankle instability, and (2) to quantify the impact of visual feedback on balance within this particular population. A systematic search of the Cochrane Central Register of Controlled Trials, MEDLINE, Web of Science, and PubMed databases was conducted by the first author in April 2024 following the PERSiST guidelines and PRISMA statement [33, 34]. The search strategy employed a variety of search terms and Boolean operators across the different databases, to identify relevant studies. Using Pubmed as an example, the Boolean search syntax is: (‘function ankle instability’ or ‘chronic ankle instability’ or ‘ankle sprain’ or ‘ankle injury’) AND (‘balance’ or ‘dynamic balance’ or ‘static balance’ or ‘SEBT’ or ‘FAAM’) AND (‘VR’ or ‘feedback’ or ‘visual’). The search terms used in PubMed and MEDLINE were “ALL Fields,” in Web of Science “Topic,” and in the Cochrane Central Register of Controlled Trials “ALL TEXT.” The meta-analysis was registered with INPLASY on June 16, 2024, under the registration number 10.37766/inplasy2024.6.0054. The search strategies are provided in the supplementary materials.

### Eligibility criteria and data items

The first author was responsible for the exclusion of all duplicate studies. The titles and abstracts of the remaining studies were then screened for relevance by two researchers independently. (C.W. and L.S.) Disagreements were resolved through discussion or consultation with another researcher (J.L.), and the full text was finally assessed for eligibility. To be included in the review, articles must comply with the following criteria: (1) original research studies; (2) research conducted on or likely to benefit the population with ankle instability; (3) Inclusion of English-only literature; (4) it must involve enhanced visual feedback of some type; (5) it must involve improvement of balance indicators or balance functions; (6) studies comparing visual feedback exercise therapy interventions with conventional exercise control therapies, cluster randomized trials, and randomized crossover studies were included. Literature that did not fulfill the aforementioned criteria was excluded from further consideration. Concurrently, the search was extended through the application of “forward-searching” and “backward searching” following the established criteria, thereby addressing the identified gaps in the initial search results.

Following the identification of the literature for inclusion, data on the following variables were collected and reported by two researchers for each of the included studies. These variables included the authors, title, years, study design, participants, sex, visual feedback types, feedback time, exercise protocols, outcomes, balance ability, and motor function. The data were extracted as mean (M) plus standard deviation (SD). In the absence of experimental data, the analysis of images in the text was conducted using PlotDigitizer. The authors were subsequently requested to provide the data above via email in instances where it was absent from the text. A third researcher (J.L.) subsequently verified the extracted data before analysis.

### Risk of bias and certainty of evidence

The Cochrane Risk Assessment Tool was employed to evaluate the risk of bias inherent to the included articles. The assessment was conducted by two independent reviewers (C.W. and L.S.), and any discrepancies were resolved through consensus or, if necessary, by a third reviewer (J.L.).We used The Grading of Recommendations Assessment, Development, and Evaluation (GRADE) method to assess the quality of evidence for different types of visual feedback for balance rehabilitation in people with ankle instability, with disagreements resolved by a third reviewer (J.L.) [35].

### Effect measures and synthesis methods

To analyze the benefits, we calculated effect sizes in individual studies as mean differences(MD) and standard deviation(SD), thus enabling the combination and comparison of outcomes assessed in individual trials. A meta-analysis was conducted using Review Manager 5.4.1 and STATA 18.0. The combined effect sizes were reported with 95% confidence intervals and 95% prediction intervals. A 95% confidence interval that does not cross zero indicates a statistically significant result. We conducted sensitivity analyses using STATA 18.0 to evaluate the potential influence of including studies with significantly heterogeneous outcomes on the combined results. To assess the potential for publication bias, we generated funnel plots using Stata 18.0 and performed Begg’s test and Egger’s regression tests. A *p*-value of less than 0.05 was deemed indicative of statistically significant publication bias.

To quantify the acute effect of visual feedback on balance, an analysis was conducted on balance data obtained from the Biodex Balance System, the results of the Star Excursion Balance Test (SEBT), and the Foot and Ankle Ability Measure (FAAM) [36]. The Biodex system is utilized to assess the subject’s capacity to maintain equilibrium and stability in three primary planes of movement (anteroposterior, mediolateral, and overall) on an unstable inclined platform. This is achieved by regulating the angle of inclination of the platform. The balance test is classified according to the following categories: static balance, dynamic balance level 2, dynamic balance level 4, and dynamic balance level 8 [37]. In clinical practice, the SEBT is a recognized and reliable method for assessing the effectiveness of interventions related to stability [38]. The FAAM is a functional assessment tool used to evaluate the stability of the ankle joint. Higher scores on the FAAM reflect superior self-reported function. Balancing capacity measurements from the Biodex Balance System are presented, where lower values indicate enhanced balancing ability. A data conversion process was conducted during the analysis phase to ensure compatibility with the subsequent meta-analysis.

To compare the effects of visual feedback on balance ability, the pre and post-performance changes in the feedback and control groups were utilized as a basis for comparison. The mean of the M(change) was calculated as follows: The difference between the mean values for the post-test and the pre-test was calculated as M(post) - M(pre). The standard deviations (SD) for the pre and post-tests were calculated using the formulas outlined in the Cochrane Handbook for Systematic Reviews of Interventions [39]. As the included studies did not report correlation values, the correlation coefficient was set at *r* = 0.5. In instances where data were presented as standard deviation plus standard error (SE), the Cochrane Handbook calculation tool was employed to convert the SE to SD.

A meta-analysis of the transformed data was conducted using the random effects model. Additionally, a sensitivity analysis was performed when I² was greater than 50% to investigate potential sources of heterogeneity. In instances where outlier effects were identified within the data set, an investigation was conducted to ascertain whether the observed heterogeneity remained acceptable following the removal of the identified outliers. If the observed heterogeneity is deemed to be within an acceptable range, models that have been adjusted to remove any outlier effects are retained.

## Result

### Study selection

**Fig. 1:**
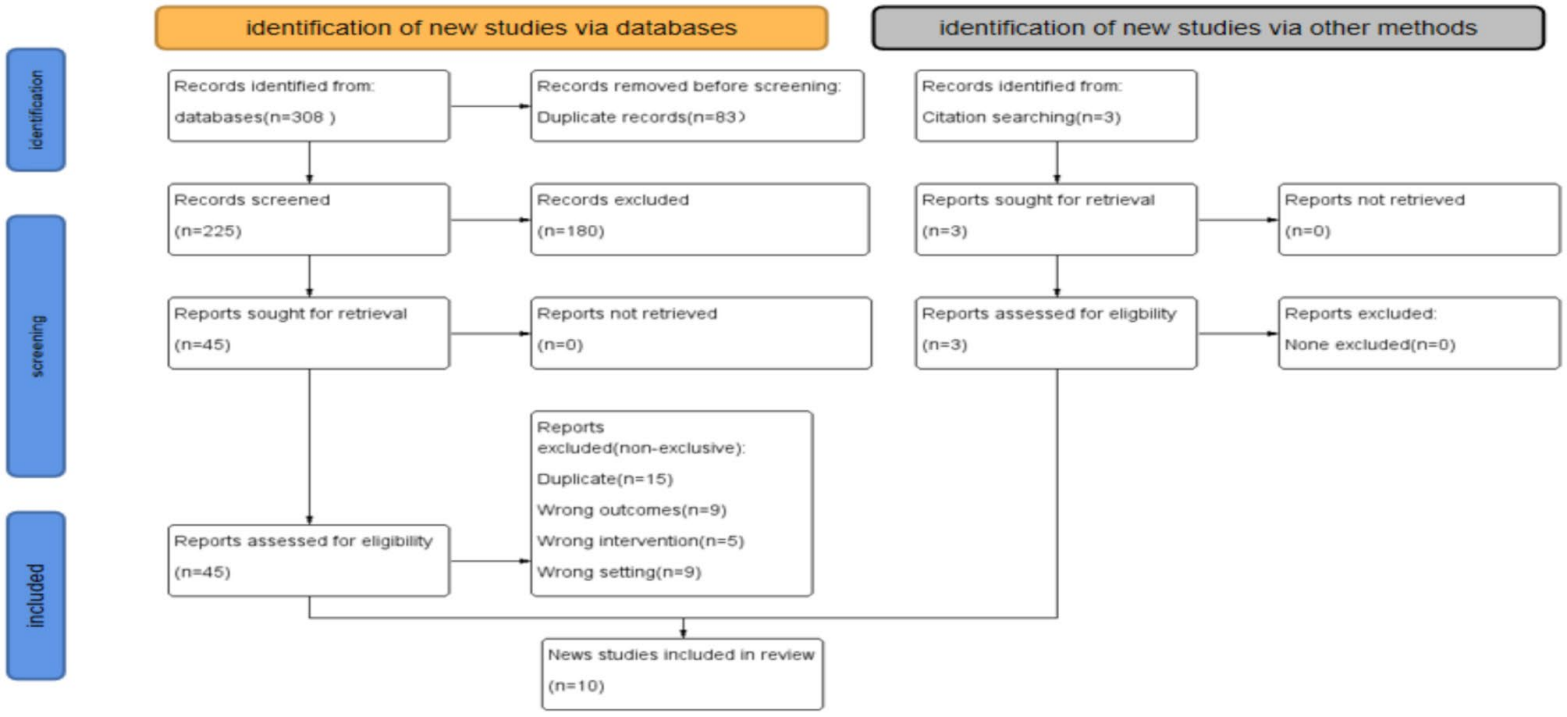
PERSiST flow diagram detailing inclusion and exclusion of manuscripts

A systematic search yielded a total of 308 studies. Of these, 83 were removed due to duplication. The titles and abstracts of the remaining 225 studies were meticulously reviewed and screened, leading to a comprehensive evaluation of 45 documents. Additionally, three further studies were identified through the reference screening process. Upon completion of the full-text review, a total of ten studies were found to meet the established inclusion criteria. The methodology for the search and screening process is depicted in Fig. 1.

### Study characteristics

This systematic review and meta-analysis includes ten studies, of which nine specifically assess the effects of visual feedback training on individuals with ankle instability. One study also explored the potential benefits of visual feedback for healthy college students, which may be applicable to patients with ankle instability (refer to Table 1) [40]. In total, the analysis encompasses 415 participants, comprising 135 females and 253 males. Among the studies, seven included both male and female subjects, two focused exclusively on male participants, and one did not specify the gender of the subjects [32].

In discussing the provision of visual feedback, five studies employed virtual reality devices, four studies utilized electronic screens, and one study focused on an external method to enhance visual feedback. Among the virtual reality devices, the Nintendo Wii Fit Plus is particularly noteworthy, as it offers guided and completed balancing exercises through a virtual program that provides visual feedback on participants’ movements during these exercises [30, 31]. The feedback delivered through electronic screens is more varied. For instance, a therapeutic exercise video game system presents human balance information on a computer screen, which is designed to assist users in completing game tasks and consequently provides visual feedback [28, 41]. Additionally, a motion capture system is utilized to visualize data related to human balance exercises, presenting this feedback to the user [32, 42]. In contrast, visual feedback was further enhanced through vestibular-ocular reflex training, which helps to replace somatosensory cues and diminish the risk of ankle injuries [40].

All study protocols incorporated a training intervention period of four to six weeks. Furthermore, the balance exercise protocols employed across these studies were not uniform, typically comprising variations such as single-leg balance exercises, lateral leg raises, lunges, and fundamental strength exercises. In addition, two studies specifically compared the effects of visual feedback training with traditional balance exercises or physical therapy on balance capability.

**Table 1:** Characteristics of the included studies

Authors, Year	Study characteristics	Visual Feedback types	Feedback exercise protocols	outcome
Fitzgerald et al. 2010 [41]	*N* = 22 healty college student (12f, 10 m) EG (*n* = 11), Age:25.4 ± 2.1 CG (*n* = 11), Age:26.9 ± 3.2 4 weeks, 3×/weeks, 15 min	Screen feedback (Active Video games)	Wobble board	SEBT, DPSI, SMI, IMI
Kim et al.2015 [30]	*N* = 20 FAI (16f,4 m) Age:23.3 ± 2.4 BEG (*n* = 10) SEG (*n* = 10) 4 weeks, 3×/weeks, 30 min	VR feedback (the Nintendo Wii Fit Plus)	Soccer heading, Ski slalom, Tight rope walk, Table tilt, Snowboard slalom.	The Biodex Balance System
Punt et al. 2016 [31]	*N* = 90 AI(39f,51 m) Wii Fit (*n* = 30), Age:34.7 ± 10.7 PTG (*n* = 30), Age:34.7 ± 11.3 CG(*n* = 30), Age:33.5 ± 9.5 6 weeks, 2×/weeks, 30 min	VR feedback (the Nintendo Wii Fit Plus)	ski slalom, penguin slide, table tilt, and balance bubble.	FAAM, VAS, Self-reported satisfaction
Kim et al. 2019 [43]	*N* = 21 FAI(16f,5 m) Age:21.0 ± 1.2 VRG (*n* = 10) CEG (*n* = 11) 4 weeks, 3×/weeks, 30 min	VR feedback (the Nintendo Wii Fit Plus)	Soccer heading, Ski slalom, Tight rope walk, Table tilt, Snowboard slalom.	The Biodex Balance System
Mohammadi et al. 2020 [44]	50 FAI (50 m) VRG (*n* = 25), Age = 22.16 ± 1.95 CG (*n* = 25), Age = 23 ± 2.57 4 weeks, 3×/weeks, 30 min	VR feedback (the Nintendo Wii Fit Plus)	Soccer heading, Ski slalom, Tight rope walk, Table tilt, Snowboard slalom.	Hop TEST
Koldenhoven et al. 2021 [32]	27 CAI GBF (*n* = 13) NBF (*n* = 14) 4 weeks, 2×/weeks, 60 min	Screen vision feedback	Intrinsic Foot Muscle Exercises, Ankle Exercises, Hip Exercises, Balance Exercises, Functional Exercises.	IC, FAAM, TSK, GROC, ROM
Forsyth et al. 2021 [42]	15 CAI (7f,8 m) VG (*n* = 9), Age:28 ± 9 NVG (*n* = 6), Age:29 ± 14 4 weeks, 1×/2weeks	Screen vision feedback	single leg balance with hip flexion, single leg stand and reach, lunge with distractive techniques, leap game.	SEBT, CAIT, PACES
Chuadthong et al. 2023 [28]	60 CAI(35f,25 m) Age: 10 ± 2 AVG (*n* = 30) CG (*n* = 30) 4 weeks, 3×/weeks, 30 min	Screen feedback (Active Video games)	Catching Fish and Russian Block video games.	SLST, FAAM, IMI
Cerbezer et al. 2023 [40]	20 CAI(10f,10 m) Age: 24.45 ± 4.89 VOG (*n* = 10) NG (*n* = 10) 4 weeks, 3×/weeks, 60 min	Vision feedback	single-leg stance, strength, single-leg hop, single-leg ball catch, unanticipated hop to stabilization.	SEBT, FAAM
Shousha et al. 2023 [45]	90 CAI (90 m) VRG *n* = 30), Age = 15.7 ± 0.95 BBTG (*n* = 30), Age = 15.32 ± 0.92 CG (*n* = 30), Age = 15.23 ± 0.84 3 month, 3×/weeks, 30 min	VR feedback (the Nintendo Wii Fit Plus)	Soccer heading, Ski slalom, Tight rope walk, Table tilt, Snowboard slalom.	The Biodex Balance System, CAIT

FAI: Functional Ankle Instability, CAI: Chronic Ankle Instability, SEBT: star excursion balance test., IMI: Intrinsic Motivation Inventory, SMI: self-motivation inventory, IC: initial contact, FAAM: Foot and Ankle Ability Measure, VAS: visual analog scale, TSK: Tampa Scale of Kinesiophobia, GROC: Global Rating of Change, ROM: diminished range of motion, CAIT: Cumberland ankle instability tool, PACES: Physical Activity Enjoyment Scale, SLST: the single-leg stance test

### Risk of bias in studies and certainty of evidence

**Fig. 2:**
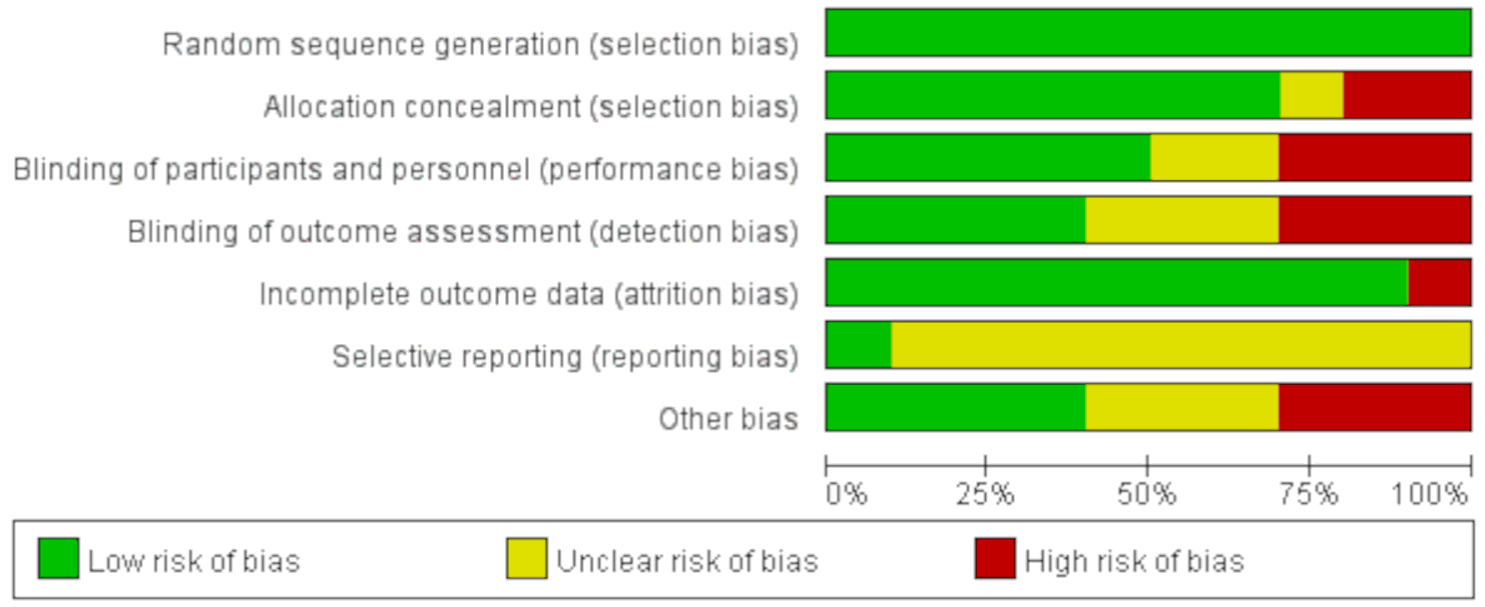
The risk of bias graph

**Fig. 3:**
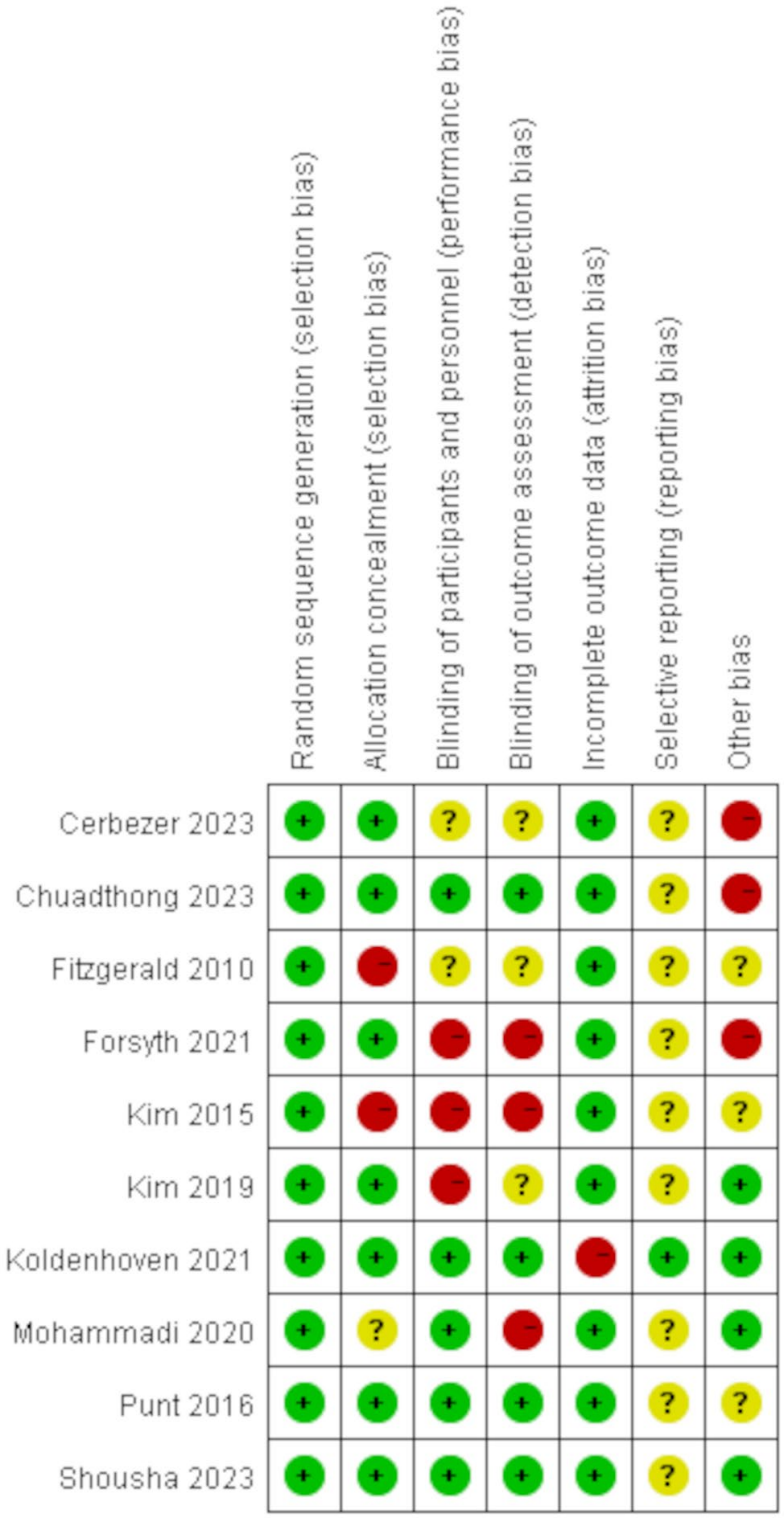
The risk of bias summary

The Cochrane Risk Assessment Tool was also used to evaluate the risk of bias table for the included articles (Figs. 2 and 3). All studies demonstrated a low-risk rating in random sequence generation risk. Two studies (20%) demonstrated high risk in allocation concealment [30, 41]. Due to the specific nature of some visual feedback, the overall blinding process has a high risk of bias (33%) [30, 32, 42, 43]. In addition, one study (10%) was found to have a high risk of bias in the incomplete outcome data [32]. Three studies (33%) did not report potential conflicts of interest, indicating a high risk of bias [28, 40, 42]. We categorized the rating of direct evidence as ‘high, medium, low, and very low’ and if any of the following occurred in the study, the GRADE rating was lowered from a high level of certainty to very low. For example: (1) In risk comparisons, more than 50% of the included studies had an ‘unclear risk of bias’ or ‘high risk of bias’. (2) Determine whether the study population, intervention, and outcome are directly related to the results of the meta-analysis by comparing study characteristics. (3) Examine the 95% CIs of the meta-analysis comparisons to determine the effect sizes of the different intervention effects; if some 95% CIs are affected by insufficient sample size, downgrade by one level. (4) To test for publication bias, we used Egger’s test and Begg’s test to test for the potential risk of publication bias using STATA software, and we generated funnel plots. According to the results of the evaluation, the final GRADE rating for this study was “medium” quality.

### Meta-analysis results

**Fig. 4:**
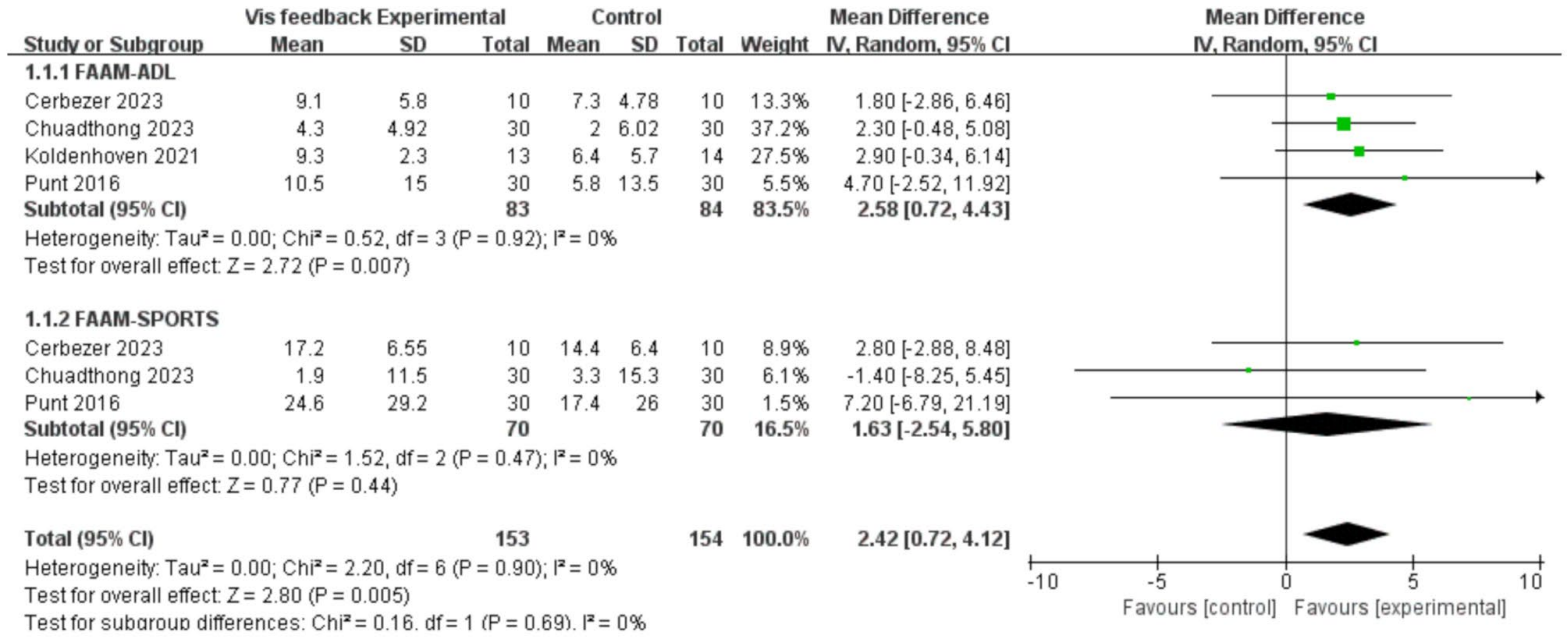
Forest plot demonstrating the chronic effects of visual feedback training on Foot and Ankle Ability Measure (FAAM) 0.95%CI:95% confidence limit, SD: standard deviation

**Fig. 5:**
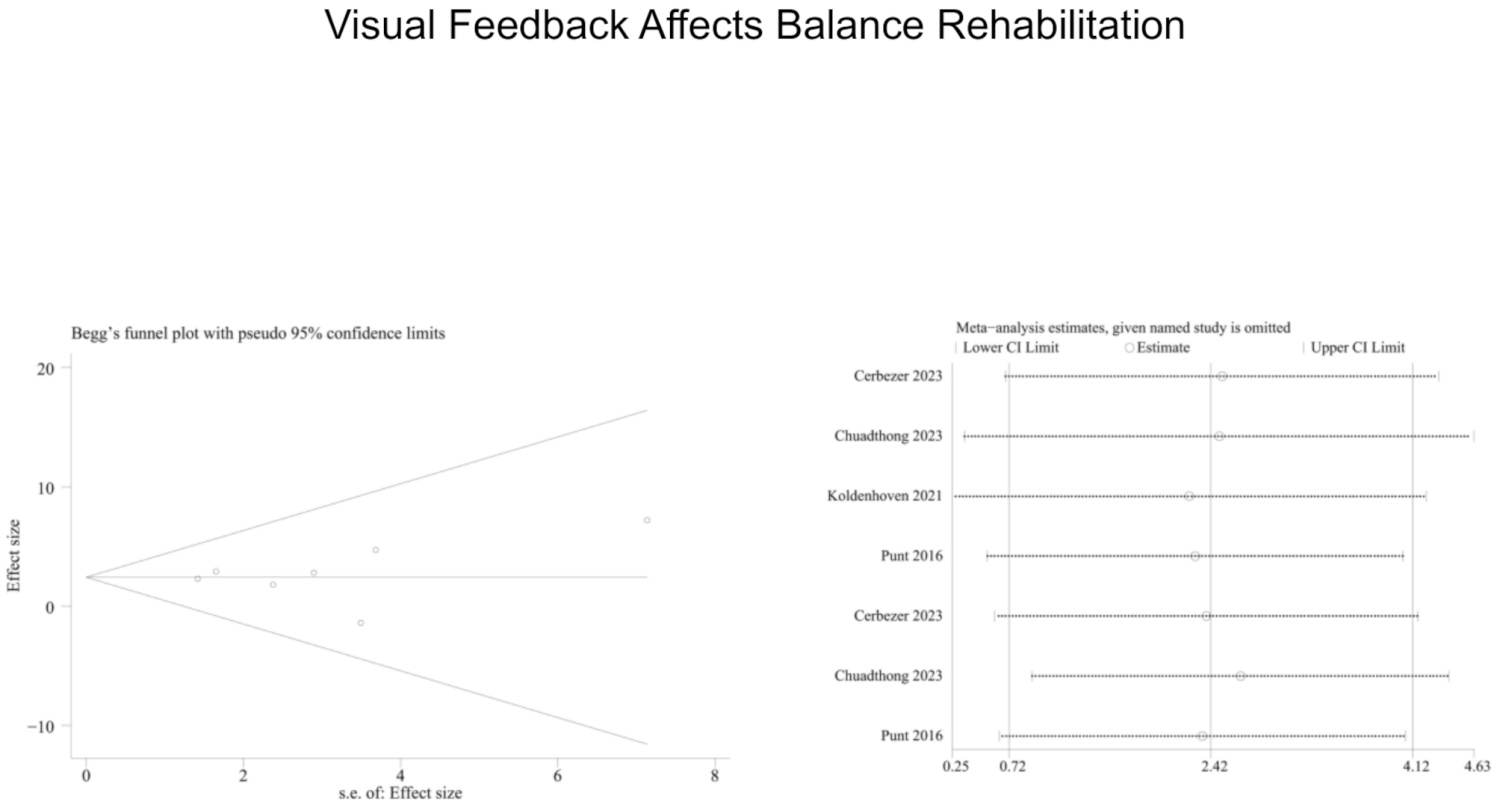
Sensitivity analysis of the chronic effects of visual feedback training on Foot and Ankle Ability Measure(FAAM)

Concerning the foot and ankle ability measure, a more pronounced effect was observed in the visual training group (MD = 2.42, 95% CI = 0.72 to 4.12, I² [total] = 0%). Among the various forms of training, visual feedback training was found to have a significant beneficial effect on FAAM-ADL (MD = 2.58, 95% CI = 0.72 to 4.43, *p* = 0.007), while no such effect was observed for FAAM-SPROTS (*p* = 0.44). (Fig. 4). Second, the sensitivity analysis and funnel plot (Begg’s test) results for the FAAM data showed that there was no significant risk of publication bias (Fig. 5).

**Fig. 6:**
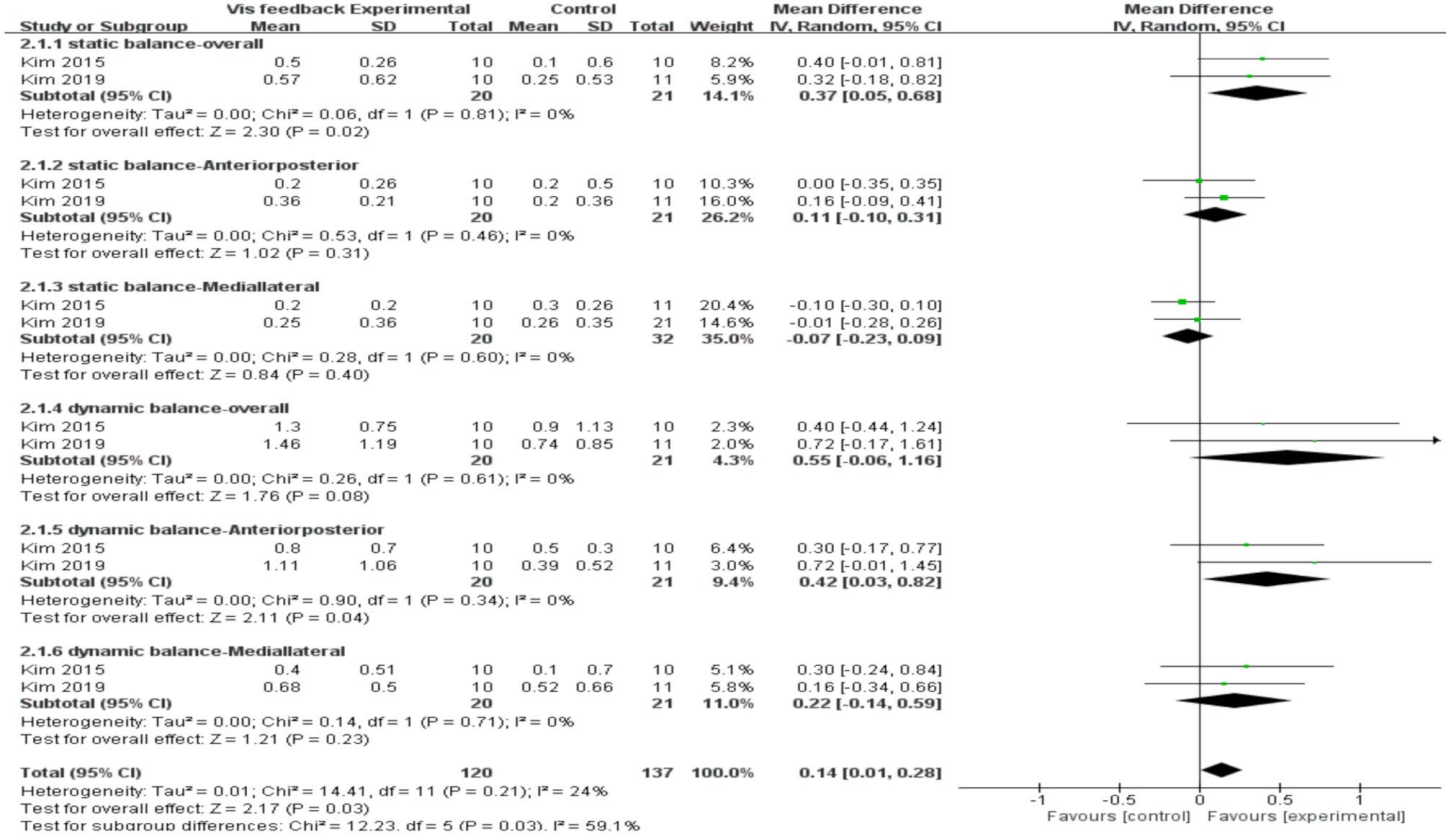
Forest plot demonstrating the chronic effect of visual feedback training on balance as measured by the Biodex Balance System.95%CI:95% confidence limit, SD: standard deviation

The Biodex Balance System data revealed a more pronounced impact for the visual training group (MD = 0.14, 95% CI = 0.01 to 0.28, I²[total] = 24%). Significant effects were demonstrated in dynamic balance tests (MD = 0.35, 95% CI = 0.11 to 0.60, *p* = 0.005). Conversely, no significant effect was observed in the static balance test (MD = 0.07, 95% CI = -0.08 to 0.22, *p* = 0.34) (Fig. 6).

**Fig. 7:**
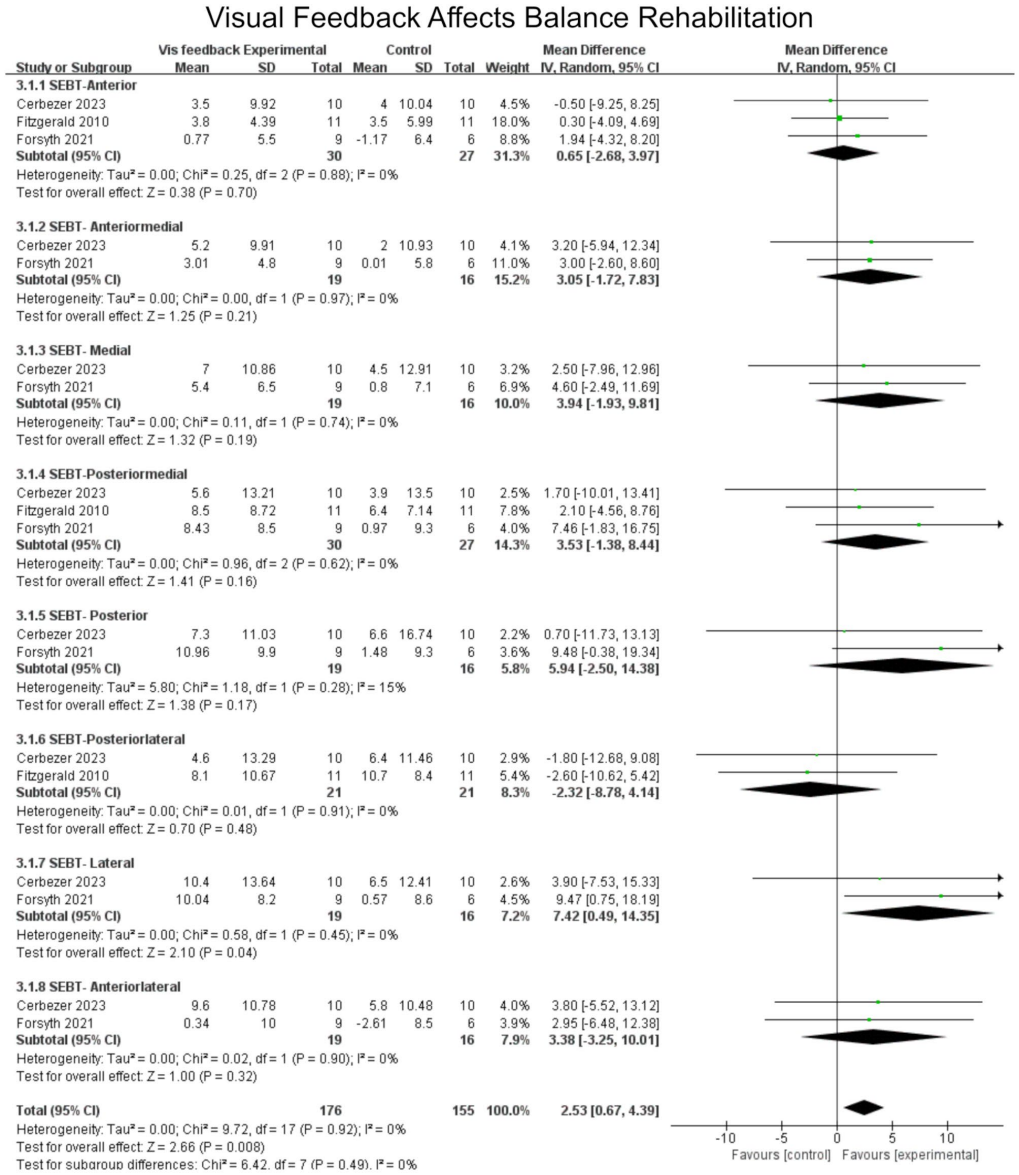
Forest plot demonstrating the chronic effects of visual feedback training on the STAR EXCURSION BALANCE TEST (SEBT)

The star excursion balance test revealed a more pronounced effect for the visual training group (MD = 4.83, 95%CI = 3.09 to 6.56, I²[total] = 21%). Of greater consequence are the posterior-medial (MD = 6. The posterior-medial (MD = 6.85, 95%CI = 1.18 to 12.53, *p* = 0.02), posterior-lateral (MD = 6.85, 95%CI = 2.68 to 10.15, *p* = 0.0008), and lateral (MD = 7.42, 95%CI = 0.49 to 14.35, *p* = 0.04) directions demonstrated a statistically significant effect (Fig. 7).

## Discussion

The objective of this systematic review and meta-analysis was to thoroughly evaluate the impact of visual feedback on balance exercises in individuals with ankle instability. Specifically, we aimed to understand how visual feedback influences balance in this demographic. Our review included ten researchers who met the inclusion criteria, and the results indicate that visual feedback provided through virtual reality devices, electronic screens, and external focus significantly enhances balance exercises for those with ankle instability. Participants showed improved performance in the Star Excursion Balance Test (SEBT), BIODEX balance assessments, and the Foot and Ankle Ability Measure (FAAM). These findings are further supported by the meta-analysis, which demonstrates that individuals who received visual feedback during balance exercises experienced enhanced balance capabilities and long-term benefits.

Due to the diverse indicators present throughout the research, we chose to concentrate on three specific variables to quantify changes in balance ability. As for the other variables, employing active video games as a rehabilitation tool for pediatric patients with ankle instability may enhance performance on the single-leg stand test (SLST) [28]. The Wii Fit had a notable positive impact on the figure-of-8 hop test and side hop performance of basketball players [44]. The PUNT study found that the Wii Fit did not lead to significant improvements in balance for individuals with Grade 1 or 2 ankle sprains. However, the data from their research does support the effectiveness of visual feedback in enhancing foot and ankle capabilities. Therefore, it can be hypothesized that most acute ankle sprains may not require specialized rehabilitation. Visual feedback appears to have a more positive impact on functional stability and chronic ankle instability resulting from sprains [31]. Furthermore, the incorporation of visual feedback has been shown to enhance the enjoyment of exercise and to foster intrinsic motivation to exercise [40, 42]. In conclusion, incorporating visual feedback into the rehabilitation process for patients with ankle instability has proven to be an effective adjunctive strategy, promoting positive long-term adaptation. To date, no research has indicated any adverse effects associated with this approach. Therefore, rehabilitation practitioners and athletic trainers should consider utilizing visual feedback as a valuable supplement to or alternative to traditional rehabilitation methods for individuals experiencing ankle instability.

### Visual feedback type

In terms of the type of visual feedback, three studies employed virtual reality devices, specifically the virtual exercise program provided by the Nintendo Wii Fit™, to provide information on the quality of movement and the results of movement execution, primarily through the completion of exercise games [46]. Four studies employed on-screen feedback to provide immediate feedback on the outcomes of human movement through the use of motion capture cameras, motion tracking devices, and motion position sensors [47]. These include a wobble board-based extracorporeal play therapy system and an active video screen game, in which the primary objective is to achieve game objectives by integrating visual and postural control to collectively direct the movement of virtual objects on the screen [28, 41]. In addition to various devices, including motion capture cameras and instrumented running tables, the system incorporates visualization software and other components that collectively generate real-time kinematic data. This data is then utilized to provide visual feedback during balance rehabilitation training [32, 42]. Another study employed a vestibular-ocular reflex training (VOR) plus neuromuscular exercise approach, wherein vision was controlled and an external focal point was utilized to enhance the synergistic work of the visual, vestibular, and somatosensory systems. These systems work in concert to improve postural control and balance through exercise [40]. This review does not address the comparative efficacy of different types of visual feedback in the rehabilitation of individuals with ankle impairments. The majority of studies have reported that the visual feedback training group exhibited outcomes that surpassed those of the training group that did not utilize visual feedback.

### Balance exercise response

A total of ten studies were conducted to explore the impact of visual feedback on the effectiveness of balance exercises. In terms of assessing participants’ abilities, four studies utilized the FAAM questionnaire, three employed the SEBT test, three used the Biodex system test, and two focused on the SLST and Hop tests, respectively. The current literature suggests that the provision of visual feedback does not diminish the effectiveness of balance exercise training compared to control groups that underwent traditional training methods. One study specifically investigated the long-term effects of balance exercises six months after the completion of vestibular-ocular reflex training, demonstrating that the benefits of the intervention were still apparent after this period. This indicates that integrating varying levels of visual feedback into rehabilitation training for individuals with ankle impairments may serve as an effective and sustainable long-term strategy for enhancing overall balance.

The available evidence suggests that visual feedback is an effective method for improving FAAM, as demonstrated by the results of studies that have assessed the FAAM questionnaire. However, there is still controversy regarding the specific effects of visual feedback on FAAM. The reason for this discrepancy is that the FAAM consists of two distinct subscales: a 21-item activities of daily living (ADL) subscale and an 8-item sports-related subscale. The results of the meta-analysis indicated that the increase in visual feedback for FAAM was primarily attributable to FAAM-ADL (MD = 2.58, 95% CI = 0.72 to 4.43, *p* = 0.007), while there was a slight but non-significant increase observed for FAAM-SPORT (*p* = 0.44) [28, 31, 40]. These findings are consistent with those of previous studies which have indicated that visual feedback may have enhanced the degree of foot and ankle involvement in subjects’ everyday activities (e.g., standing, ascending and descending slopes, ascending and descending stairs) while simultaneously reducing the extent of foot and ankle involvement in athletic activities (e.g., running, jumping, lateral movements) [36].

The provision of visual feedback during balance exercises was found to have a significant impact on changes in the SEBT, with a statistically more pronounced improvement when compared to training conducted without the inclusion of visual feedback (MD = 2.53, 95% CI: 0.67 to 4.39). Three studies analyzed performance in three directions, while two studies analyzed performance in eight directions. The results of the meta-analysis demonstrated that the SEBT-lateral effect was statistically significant in specific directions (MD = 0.42, 95% CI:0.49 to 14.35). This may be attributed to the absence of visual feedback from the posterior aspect of the body, necessitating greater reliance on somatosensory feedback, which in turn increases the demands on the body’s ability to maintain balance [48]. The enhancement of visual feedback data markedly enhances this limitation, enabling subjects to attain superior training outcomes in the SEBT by attaining greater distances in more challenging directions [29]. An additional potential explanation is that the visual feedback provided by electronic devices constitutes augmented feedback. The external focus of attention provided enables participants to redirect their attention to the impact of the exercise on the surrounding environment. A focus on the outcome of the task rather than on the physical movement can prove an effective method of improving performance during training [49–51].

Among all balance exercises assessed through the Biodex system, visual feedback from the Wii Fit had a significant effect on balance improvement (MD = 0.27, 95% CI = 0.11 to 0.43). The improvement in dynamic balance ability (MD = 0.72, 95% CI = 0.56 to 0.88) appeared to be more significant with visual feedback than with static balance ability (MD = 0.37, 95% CI = 0.05 to 0.68). This effect may be attributed to the selection of Wii Fit games (including soccer heading, ski slalom, tightrope walking, table tilting, and snowboard slalom), which encompassed a multitude of movements in various directions, including front-back, left-right, inside-outside, and overall direction.

### Visual feedback on positive motivation

Prior research has demonstrated that virtual reality (VR) devices can enhance the enjoyment and motivation associated with exercise [51, 52]. Four studies have assessed the subjective experience of providing visual feedback during training. Concerning video screen feedback, two video game studies investigating intrinsic motivation for training employed the IMI and SMI questionnaires to assess the subjective experiences associated with the target activity [28, 41, 53, 54]. Concurrently, a study employed the Physical Activity Enjoyment Scale (PACES-8) questionnaire to ascertain the degree of enjoyment experienced by subjects during visual feedback training [42]. The findings indicated that rehabilitation training with visual feedback resulted in enhanced intrinsic motivation, training enjoyment, and potentially longer exercise duration in comparison to traditional rehabilitation training without visual feedback. Furthermore, an assessment of subjects’ self-reported satisfaction and perceived effectiveness using the interview method indicated that subjects in the Wii Fit group perceived the program to be similarly effective and satisfying as subjects in the traditional rehabilitation group [55]. It can thus be posited that the provision of visual feedback throughout the rehabilitative process for patients with ankle instability may prove an efficacious method of enhancing their subjective experience. This will facilitate adherence to the rehabilitation program and enhance compliance with the prescribed rehabilitation regimen [15, 56].

### Limitations and future direction

Although this is the inaugural systematic review and meta-analysis to demonstrate the impact of visual feedback on balance rehabilitation in ankle instability populations, there are still some limitations to this study. The first limitation is the relatively small number of studies investigating the impact of visual feedback on populations with ankle instability. Additionally, the assessment metrics varied, which constrained our ability to comprehensively assess the effects of visual feedback on all relevant metrics. This raises questions about the comprehensiveness of the meta-analysis results. To address this limitation, we conducted a systematic evaluation of non-meta-analyzed metrics intending to provide a more comprehensive assessment of the effects of visual feedback. Second, regarding the results of the ‘selective reporting’ assessment, only one of the articles we assessed reported completeness of outcome data and was assessed as ‘low risk’, while the rest of the articles did not explicitly account for completeness of outcome data and reporting of missing data in the text. Therefore, we identified this as an indefinite risk, which requires readers to treat our findings with a higher degree of caution.

The first direction for future research is the identification of different sources of visual feedback. In this review, the primary focus is on integrated devices based on a combination of virtual reality equipment, motion capture cameras, and visualization software, as well as vestibular-ocular reflex training that fosters external focus. It would be beneficial to gain a deeper understanding of the sources of visual feedback, and further research is needed to identify any additional sources of visual feedback. Secondly, paired, dose, and network meta-analysis methods can be used to determine the specific rehabilitative effects of different types of visual feedback on populations with ankle instability and the size of the optimal visual feedback dose [57]. Finally, some of the included studies in this meta-analysis combined visual feedback with an exercise intervention (e.g., Wii Fit games), and some of the control groups included either a no-intervention control or a placebo control. Future studies should include a separate exercise intervention as part of the control group to determine the true effect of visual feedback.

## Conclusion

A systematic review and meta-analysis demonstrated that the provision of visual feedback during balance rehabilitation in populations with ankle instability yields substantial benefits in enhancing ankle functional sensation and balance. The review of the literature revealed that the provision of visual feedback resulted in enhanced balance and ankle function, exceeding the outcomes observed in the absence of visual feedback. Moreover, there were no reports of adverse effects. Furthermore, the provision of visual feedback during rehabilitation has been observed to exert a beneficial influence on the subject’s subjective experience, including an increase in intrinsic motivation and enjoyment of the exercise. This may have a beneficial effect on adherence to the rehabilitation program, which requires further investigation. The results of the meta-analysis indicate that, among the selected balance measures, visual feedback may be more effective than static balance in improving dynamic balance. This may be related to the specific exercise movements used. In light of these findings, it seems reasonable to suggest that the provision of visual feedback may be a beneficial addition to the rehabilitation of patients with ankle instability.

## Electronic Supplementary Material

Below is the link to the electronic supplementary material.

Supplementary Material 1

Supplementary Material 2

## Data Availability

Wang, Chuan (2024), “The Effect of Visual Feedback on Balance Rehabilitation in People with Impaired Ankle Instability: A Systematic Review and Meta-Analysis”, Mendeley Data, V1, doi: 10.17632/hddjvhvs9s.1.
